# Tunable Stochastic Pulsing in the *Escherichia coli* Multiple Antibiotic Resistance Network from Interlinked Positive and Negative Feedback Loops

**DOI:** 10.1371/journal.pcbi.1003229

**Published:** 2013-09-26

**Authors:** Javier Garcia-Bernardo, Mary J. Dunlop

**Affiliations:** School of Engineering, University of Vermont, Burlington, Vermont, United States of America; University of Tokyo, Japan

## Abstract

Cells live in uncertain, dynamic environments and have many mechanisms for sensing and responding to changes in their surroundings. However, sudden fluctuations in the environment can be catastrophic to a population if it relies solely on sensory responses, which have a delay associated with them. Cells can reconcile these effects by using a tunable stochastic response, where in the absence of a stressor they create phenotypic diversity within an isogenic population, but use a deterministic response when stressors are sensed. Here, we develop a stochastic model of the multiple antibiotic resistance network of *Escherichia coli* and show that it can produce tunable stochastic pulses in the activator MarA. In particular, we show that a combination of interlinked positive and negative feedback loops plays an important role in setting the dynamics of the stochastic pulses. Negative feedback produces a pulsatile response that is tunable, while positive feedback serves to amplify the effect. Our simulations show that the uninduced native network is in a parameter regime that is of low cost to the cell (taxing resistance mechanisms are expressed infrequently) and also elevated noise strength (phenotypic variability is high). The stochastic pulsing can be tuned by MarA induction such that variability is decreased once stresses are sensed, avoiding the detrimental effects of noise when an optimal MarA concentration is needed. We further show that variability in the expression of MarA can act as a bet hedging mechanism, allowing for survival in time-varying stress environments, however this effect is tunable to allow for a fully induced, deterministic response in the presence of a stressor.

## Author Summary

Cells can sense their environment and respond to changes, however the sudden appearance of a stressor can be catastrophic if the time it takes to sense and initiate a response is slow relative to the action of a stressor. A possible solution is to couple a sensory response with a stochastic, random approach. In the absence of stress, a random subset of cells expresses resistance genes, ensuring that if a stressor appears there will be some cells that are able to survive and regenerate the population; once stress is sensed all cells should respond by expressing resistance genes. Such an approach is particularly advantageous when resistance mechanisms are taxing to the cell because it limits their expression when no stress is present. We studied this phenomenon computationally using a model of the multiple antibiotic resistance activator, MarA. MarA controls over 40 resistance genes and can be induced by many harmful compounds. We show that when uninduced, the gene regulatory network controlling MarA is capable of producing stochastic pulses that can serve to hedge against sudden changes in the environment with minimal cost to the population. When induced, MarA expression is elevated and has low variability to ensure a uniform response.

## Introduction

Antimicrobial drug resistance has been studied extensively due to its clinical importance. Traditionally, research has focused on heritable genetic mechanisms, but transient mechanisms, where only a subset of the population expresses resistance genes, are beginning to receive attention for their role in the recalcitrance of chronic infections [1]. Examples of transient resistance include bacterial persistence, inducible expression of antibiotic efflux pumps, and biofilm formation [1]–[3]. Although these mechanisms can provide resistance or tolerance to a broad spectrum of chemicals, they are often taxing to the cell, slowing growth or utilizing resources [4], [5]. Importantly, transient resistance can occur within an isogenic population, where phenotypic variation can provide diversity to hedge against catastrophic events due to unpredictable fluctuations in the environment by insuring that some fraction of the population is always in a resistant state [6]–[11].

MarA, the multiple antibiotic resistance activator, is a global regulator of resistance genes. It is conserved across enteric bacteria including *Klebsiella*, *Salmonella*, *Escherichia*, *Enterobacter*, and *Shigella* species, but is best studied in *Escherichia coli* [12]. Bulk population studies have shown that MarA plays an important role in multidrug tolerance by inducing expression of over 40 genes implicated in antibiotic resistance [13]–[19]. Examples include the AcrAB multidrug efflux pump; *micF*, an antisense RNA that represses expression of the outer membrane porin OmpF; SodA, a manganese-containing superoxide dismutase; and the outer membrane channel TolC [15], [20], [21].

**Figure 1:**
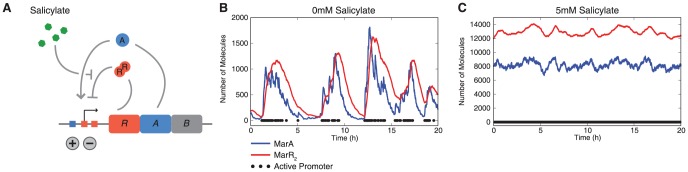
The *marRAB* operon and stochastic modeling results. (A) Schematic representation of the *marRAB* operon encoding *marR* (*R*, repressor) and *marA* (*A*, activator). MarA and two copies of the MarR_2_ dimer bind to the *marRAB* operator; salicylate and other aromatic compounds allosterically inhibit repression by MarR_2_. (B) Stochastic simulations of the uninduced *marRAB* operon show stochastic pulses in MarA and MarR_2_ protein expression. Pulses correspond to times when promoter is in the active state, i.e. one MarA and no MarR_2_ molecules are bound to the *marRAB* promoter. (C) Stochastic simulations of the *marRAB* operon induced with 5 mM salicylate. MarR_2_ levels shown in (B) and (C) include the dimeric form of the protein both with and without salicylate bound. Note the difference in y-axis scale between the uninduced and induced simulations.

Expression of MarA is inducible, providing increased resistance in response to a sensed compound. As shown in Fig. 1A, *marA* is arranged in an operon with two other genes: *marR*, the multiple antibiotic resistance repressor and *marB*, which does not play a role in regulation [14]. The *marRAB* operon is activated by monomeric MarA, which binds to a single site upstream of the −35 site, and is repressed by dimeric MarR (denoted MarR_2_), which binds to two sites, one between the −10 and −35 sites and one downstream of the transcriptional start site of the operon [14]. A variety of chemicals including phenolic compounds, uncoupling agents, redox-cycling compounds, and aromatic acid metabolites can activate transcription of *marRAB* [12], [22], [23]. Of the known inducers of *marRAB*, the weak aromatic acid salicylate is the best studied and is known to bind directly to MarR [24], [25]. Upon addition of 5 mM salicylate, transcription of *marRAB* increases 21-fold [19]. Not all genes in the *mar* regulon are activated by the same MarA concentrations, suggesting that a graded response is possible with less costly genes expressed first and more burdensome genes expressed only once high MarA levels are reached [15], [26].

The regulatory network controlling MarA consists of interlinked positive and negative feedback loops (Fig. 1A). We asked what role these opposing actions play in controlling the dynamics of MarA. Recent studies have shown that interlinked positive and negative feedback can produce a wide range of dynamic behaviors. Examples include robust oscillations, bistability, monostability, or stochastic pulsing [27]–[29]. Several synthetic oscillators have been constructed using interlinked feedback [30]–[32] and it has also been shown to be a common feature in many natural examples of biological oscillators [28]. Stochastic pulsing is emerging as an important feature in gene regulation, regulating competence, sporulation, and stress response in *Bacillus subtilis* [33]–[36], persistence in bacteria [37], [38], calcium stress response and glucose repression in *Saccharomyces cerevisiae* [39], and virulence factors in bacteria [11], [40]–[44]. More generally, phenotypic diversity within a population has been shown to increase the net growth rate under uncertain environments [6]–[11]. Although bulk population studies have demonstrated that MarA expression can be induced by inhibition of the negative feedback loop, we asked what role the opposing interlinked loops play and how these effects are manifested at the single-cell level.

To study this, we developed a stochastic model of the *marRAB* network. Our findings suggest that the interlinking of positive and negative feedback can produce stochastic pulses in MarA expression when the system is uninduced. Induction with salicylate leads to elevated levels of MarA and decreased variability. By comparing the native network with a reduced noise variant computationally, we show that stochastic pulsing can act as a bet hedging mechanism to insure that some fraction of the population is always expressing resistance genes. The combination of stochastic pulsing and inducible non-noisy expression of MarA can serve to tune the stochasticity of the system to hedge against environmental uncertainty, while allowing for a deterministic response when a stressor is sensed.

## Results

### Stochastic pulsing and noise control

We developed a stochastic model to study MarA expression dynamics. In the model, protein production is the result of a series of single random events [45], including reactions for transcription, translation, and folding of MarA and MarR, dimerization of MarR to MarR_2_, MarA and MarR_2_ association and dissociation events at the *marRAB* promoter, MarR_2_ inhibition by salicylate, and mRNA and protein degradation. Reaction rates and constants were drawn from the literature using experimentally derived values (Methods, Table S1) and simulations were conducted using the Gillespie stochastic simulation algorithm [46].

We first asked how the dynamics of MarA expression change with and without induction at the single-cell level. Bulk population studies have shown that MarA expression can be induced [19], [23], however it is not clear whether these population-level results obscure more complex dynamics in individual cells. Using a stochastic computational model, we observed distinct pulses in expression of MarA and MarR_2_ in the absence of induction (Fig. 1B). The pulses are caused by brief periods when both MarR_2_ molecules dissociate from the *marRAB* promoter and MarA binds, initiating expression of the *marRAB* genes. This is terminated when one or two copies of MarR_2_ bind to the operator, shutting down transcription, and resulting in a pulse in the expression of *marRAB* genes. Stochastic pulsing has been shown experimentally for several bacterial systems [6]–[11], [33]–[38], [40]–[44]. The phenomenon observed here is consistent with data from other well-characterized repressed systems such as the lactose [47], tryptophan [48], and arabinose [49] operons, where a transcriptional burst occurs when the repressor randomly dissociates from its binding sites.

In contrast to the uninduced system, our simulations show elevated levels of MarA expression when induced, but lack the pulsing behavior observed in the uninduced state (Fig. 1C). In the presence of a harmful compound, constant, high MarA expression would allow the cell to counteract the noxious effects of a stressor without dipping into a state of low tolerance or rising into a regime with unnecessary cost. When induced, the *marRAB* promoter spends most of the time in an active state with MarA bound and no MarR_2_ present, resulting in reduced noise and elevated expression of MarA. To further clarify the mechanism behind the pulsing behavior, we analyzed the corresponding deterministic system, finding a single stable fixed point for all values of salicylate (Fig. S1).

**Figure 2:**
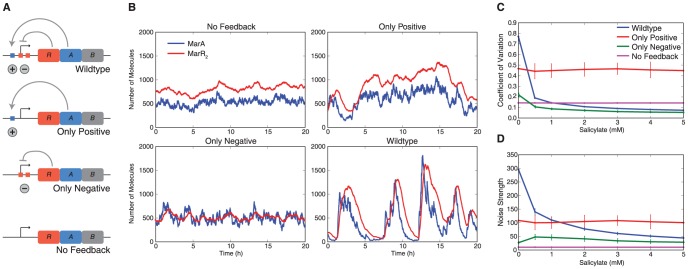
Stochastic pulsing mediated by interlinked positive and negative feedback and tuned by inducer levels. (A) Schematic representation of the four variants of the *marRAB* network studied. The *Wildtype* case has binding sites for MarA and MarR_2_, *Only Positive* eliminates both binding sites for MarR_2_, *Only Negative* eliminates the MarA binding site, and *No Feedback* has constant, constitutive expression. (B) Stochastic simulations of the four network variants. Noise amplification is observed in the *Only Positive* variant, transcriptional bursting appears in the *Only Negative* case, and both characteristics are combined to create high-amplitude stochastic pulsing in the *Wildtype* network. (C) Coefficient of variation (CV, std/mean) of MarA as a function of salicylate concentration. Constant noise is observed for the variants that do not respond to salicylate (*Only Positive* and *No Feedback*). The salicylate-responsive variants (*Wildtype* and *Only Negative*) show a decrease in CV upon induction. (D) Noise strength (var/mean) of MarA as a function of salicylate. Error bars in (C) and (D) show standard deviation across 100 replicates.

We hypothesized that the stochastic pulses in MarA observed in the uninduced system were caused by the interlinked positive and negative feedback loops that control expression of the *marRAB* operon. To study the relationship between the feedback loops and noise dynamics we compared four variations on the *marRAB* operon model (Fig. 2A): (i) *Wildtype*, which includes the complete operator with all binding sites intact; (ii) *Only Positive*, which eliminates both MarR_2_ binding sites, leaving only the positive feedback loop; (iii) *Only Negative*, which eliminates the MarA binding site, leaving the negative feedback loop; and (iv) *No Feedback*, which removes both feedback loops so that the *marRAB* operon is constitutively expressed. To allow for a controlled comparison between the four networks, we fixed the mean expression of MarA such that it was the same for all networks when the systems were uninduced.

Our findings show that stochastic pulsing is the result of the interaction between the positive and negative feedback loops. We tested the four network variants to quantify how the individual loops influenced the dynamics of MarA (Fig. 2B). In the *No Feedback* variant expression is constitutive and low levels of noise come from small fluctuations in the birth and death of mRNA and proteins. In the *Only Positive* case the random fluctuations in MarA levels are amplified. Random increases in MarA lead to further elevated levels of MarA due to positive feedback, while fluctuations that decrease protein levels lower the probability of expression, leading to slow fluctuations in MarA expression. In the *Only Negative* variant we observe transcriptional bursting when both MarR_2_ molecules dissociate from the promoter, but because the system lacks positive feedback, bursts in expression are not amplified. Finally, in the *Wildtype* variant transcriptional bursts created by negative feedback are amplified by positive feedback, since MarA levels increase faster than MarR_2_ levels [19] and the presence of MarA decreases the apparent binding rate of MarR_2_ [16], likely due to steric hindrance [50]. Thus, stochastic pulsing is caused by the combination of positive and negative feedback loops, where the negative feedback loop produces pulses and the positive feedback loop serves to amplify them.

To analyze the contributions of the two feedback loops in the presence of increasing levels of the inducer salicylate, we measured the coefficient of variation and noise strength of MarA for each system (Figs. 2C and D). The coefficient of variation (CV) is the standard deviation divided by the mean. It measures the relative variation in the system, however decreases in CV can be the result of either decreased noise or increased mean [7]. Therefore, we also considered noise strength as a measure of variability. Noise strength is defined as the variance divided by the mean (also known as the Fano factor); higher noise strengths imply that the variability is high relative to the mean, giving a sensitive measure of noise [45]. As salicylate is added, mean MarA levels go up in the variants with negative feedback (Fig. S2). For the systems without negative feedback both CV and noise strength are independent of salicylate concentration.

The combination of positive and negative feedback amplifies noise in the absence of induction, while allowing for tunable noise levels. Histograms of MarA expression for the four network variants show that the *Wildtype* system produces a long-tailed distribution of MarA, while none of the other networks show this behavior (Fig. S3). This subpopulation of cells with high MarA levels will induce resistance mechanisms, which can hedge against the sudden appearance of a stressor. In the *No Feedback* and *Only Positive* variants, both CV and noise strength are constant, with positive feedback leading to higher noise (Figs. 2C and D). Interestingly, in the *Only Negative* case, the CV level depends upon induction, while noise strength does not. This is because salicylate produces a reduction in active MarR_2_ levels, which is equivalent to a reduction in the MarR_2_-promoter association constant k_r_. This parameter is independent of noise strength for a wide range of values in negative autoregulation [51]. The decrease in *Wildtype* variability observed in Figs. 2C and D arises from a disruption of stochastic pulsing and is not solely the result of an increase in MarA levels as the system is induced.

### Stability, noise, and feedback strength

We next asked if the *Wildtype* dissociation constants we derived from the literature place the system in a favorable regime that minimizes the cost of expressing burdensome resistance machinery while maximizing the chance of survival in an uncertain environment. To study this, we tested a range of association rates for MarA and MarR_2_ promoter binding while keeping the dissociation rate fixed and calculated both the cost of expressing MarA and the noise strength of MarA.

**Figure 3:**
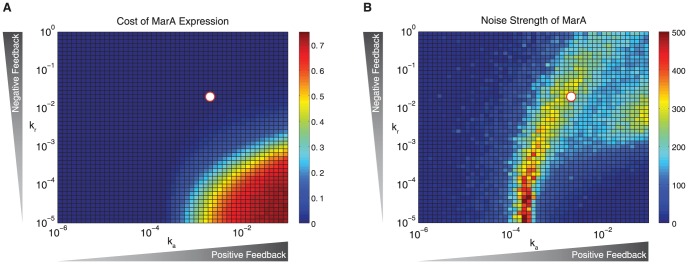
Positive and negative feedback strengths place the system in a low cost, high noise regime. (A) Cost of MarA expression as a function of the activator and repressor association rates, k_a_ and k_r_. (B) Noise strength (var/mean) as a function of k_a_ and k_r_. White circles in (A) and (B) show the nominal *Wildtype* system parameters. Data show mean values of five replicates.

MarA induces many genes within the *mar* regulon that provide resistance to stressors, but expression of these genes is taxing to the cell [4], [5], [15]. We calculated the cost of MarA expression by using the experimentally-derived function from [5], which gives cost as a function of salicylate. We related salicylate levels from this function to MarA expression directly by using data from previously published studies [5], [12] (Fig. S4, Text S1). Increased positive feedback and decreased negative feedback strengths, given by association rates k_a_ and k_r_, produce higher levels of MarA, which result in a higher cost. By this metric, the *Wildtype* network is in a very low cost regime (Fig. 3A).

We also calculated the noise strength as a function of the association rates, showing that a region of high noise strength exists when the association rates of the activator and repressor are balanced (Fig. 3B). The nominal feedback strengths of the *Wildtype* system place the system on a plateau of high noise strength, guaranteeing stochastic pulsing and relative insensitivity to feedback strength. The curvature of the elevated noise strength regime is due to the nonlinear nature of the interactions between the binding of MarA and MarR_2_ to the promoter. The high noise strengths observed when the MarR_2_–promoter association rate k_r_ is low are the result of very slow fluctuations in MarA and MarR_2_ that keep the system far from the mean. The *Wildtype* system is in a region with low cost and high noise strength. This combination of conditions enables the creation of MarA pulses, which can trigger the induction of antibiotic resistance genes without undue burden to the population.

### Parametric sensitivity analysis

To study the robustness of our results, we conducted a sensitivity analysis for all model parameters to ensure that our findings were not specific to a particular set of values. For equivalent systems with 2-fold increases and decreases relative to the wildtype parameters, we calculated the number of MarA pulses and the noise strength of MarA and compared them to the results observed in the original system (Fig. S5). In all cases, results mirrored those from the original system with pulses in MarA observed with 0 mM salicylate but not with 5 mM salicylate. Additionally, we calculated the noise strength for MarA, which showed similar results: noise strength is higher in the absence of salicylate. The sensitivity analysis provides insight into the model parameters that have the largest impact on pulsing dynamics. When the transcription, translation, or degradation rates are modified, the number of pulses and the noise strength are correlated with MarA levels. In other words, when MarA levels go up due to changes in these parameters, MarA pulse numbers and noise strength increase; decreases result when the protein levels go down.

### Noise in time-varying stress environments

Antibiotics and other harmful compounds are ubiquitous in the environments where bacteria grow, however their appearance is often non-constant and time varying. Such dynamic stress profiles have forced prokaryotes to develop mechanisms to protect themselves, including expression of pumps, superoxide dismutases, and other enzymes [52]–[54]. Cells can take several approaches when expressing resistance genes. First, they could always express the resistance genes ensuring that they will be prepared for the sudden appearance of a stressor, but the downside of this approach is that expression can be burdensome. Alternatively, cells could induce resistance genes in response to a sensed stressor. Finally, individual cells within a population could stochastically express resistance genes such that at any given time some cells in the population would be in a resistant state. Bulk population studies have demonstrated that expression of MarA and subsequent resistance is inducible. Here, we have shown computationally that in addition to this inducible resistance, expression of MarA can exhibit stochastic pulses when uninduced. We asked what benefit the combination of stochastic pulsing and inducible resistance provides to cells.

For inducible resistance mechanisms, a system must respond to a sensed signal and turn on expression of resistance genes, thus, there is a delay between the time when a stressor appears and when the response in mounted. Following induction with salicylate, maximal transcription of *marRAB* is observed after 30 minutes [55]. MarA must activate downstream genes, further delaying appearance of the resistance phenotype, as demonstrated in experiments with the MarA homolog SoxS [56]. Because expression of resistance mechanisms is not instantaneous with an inducible system, the system is vulnerable to the sudden appearance of a stressor. Stochasticity in expression of MarA in the uninduced state would allow for some fraction of cells to always be in an elevated state of resistance, ready to counter the unexpected appearance of a stressor.

We hypothesized that tunable variability would increase survival in a time-varying stress environment. To test this, we implemented a stochastic competitive growth assay to compare the fitness of the *Wildtype* network to a new variant with reduced noise (*Reduced Noise*). Competition assays can be used to discriminate between genotypes in order to identify those that achieve higher population fitness [57]–[59]. We developed a stochastic competition assay by using a modified evolutionary algorithm: cells are first initialized with equal representation of each of the alternative networks, simulation are performed, the cost for each cell is calculated, and cells with poorly performing phenotypes are replaced by top performers (Methods). To allow for a controlled comparison between the networks, we required that the *Reduced Noise* network have the same mean MarA and MarR_2_ expression as *Wildtype* for all salicylate levels and the same response time after induction with salicylate when simulations are started from the same state (Fig. S6), satisfying the equivalence requirements from [60]. The *Reduced Noise* network exhibits less variability than the *Wildtype* system, as shown in Fig. S6, due to a reduction in the MarR_2_ inhibition constants and independent binding by MarA and MarR_2_ at the promoter (Methods). Therefore, the time scale and mean levels of the induced response are identical for both variants, while the stochastic response is attenuated in the *Reduced Noise* variant.

**Figure 4:**
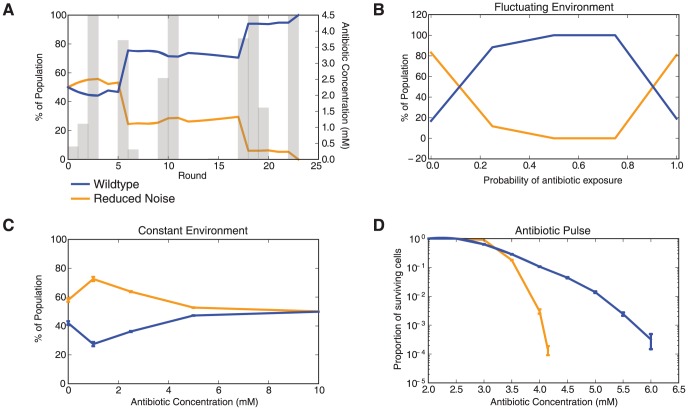
Stochastic pulsing acts as a bet hedging strategy. (A) Competitive growth simulation with *Wildtype* and *Reduced Noise* network variants. Gray bars show the antibiotic stress profile as a function of time, with heights indicating the antibiotic concentration. Antibiotic is introduced randomly with a probability of 0.5. In other words, there is a 50% chance of the antibiotic being introduced. Note that the *Wildtype* network is at an advantage when concentrations of antibiotic jump from off to high in a short time span. (B) Final proportion of *Wildtype* and *Reduced Noise* cells as a function of the probability of antibiotic exposure in a fluctuating environment. (C) Final proportion of *Wildtype* and *Reduced Noise* cells as a function of antibiotic concentration in a constant environment. Error bars show standard deviation over three runs. (D) Proportion of surviving cells after a pulse of antibiotic. Simulations are of 10,000 cells. Error bars show standard deviation over five runs.

We found that the optimal strategy for surviving antibiotic stress depends on the frequency with which the stressor appears. We first varied the probability of antibiotic addition in a time-varying stress profile (Fig. 4A). The *Wildtype* network outperforms the *Reduced Noise* network with large improvements coming when fluctuations in antibiotic levels jump from off to high in a short period of time. When high antibiotic levels are preceded by a period of low or moderate antibiotics, the *Reduced Noise* network is at a slight advantage because the resistance genes are already induced for both variants and the variability is lower in the *Reduced Noise* case. Fig. 4B summarizes the average response of the two networks as a function of the probability of antibiotic addition. For time-varying stress profiles we found that phenotypic variability allows cells with the *Wildtype* network to outperform the *Reduced Noise* variant since they are able to survive sudden, large increases in antibiotic concentration.

In contrast, we found that when we competed the *Wildtype* and *Reduced Noise* variants in a constant environment the *Reduced Noise* variant outperformed the *Wildtype* system (Fig. 4C). In a constant environment there is no advantage to having variability in MarA and rises and dips will send the system into states that are more costly or less fit. Findings from the constant environment demonstrate that the results shown in Fig. 4B are not the result of a systematic bias in favor of the *Wildtype* variant. Instead, we find that the *Wildtype* variant outperforms the *Reduced Noise* system only in fluctuating, non-constant stress environments, suggesting that variability can be helpful under certain dynamic stress profiles.

Results from the competition simulations with time-varying stress show that variability in MarA is important for surviving the sudden appearance of antibiotics. We asked whether stochastic pulses in MarA expression could be used as a bet hedging strategy by a population of cells. To test this we simulated cells for an initialization period in the absence of antibiotics and then introduced a single pulse of antibiotic, quantifying the fraction of the population that was able to survive (Fig. 4D). As the magnitude of the antibiotic pulse increases, the fraction of cells that survived decreases. However, the survival percentages depend upon how MarA expression is controlled. When antibiotic pulses are of high magnitude, the *Wildtype* populations have some cells that are in a high MarA state and are able to survive the treatment. Low amplitude pulses favor the *Reduced Noise* system because at any given time more cells are in a resistant state than with the *Wildtype* network where a larger range of MarA levels are sampled. Those cells in a low MarA state do not have enough time to mount a response when the appearance of antibiotics is sudden. Consequently, stochastic pulses help populations of cells to insure against the sudden appearance of an antibiotic where sensing-based mechanisms would be too slow to respond.

## Discussion

The analysis presented here reveals how the combination of stochastic gene expression with inducible tolerance can serve to increase population-level survival in dynamic, time-varying stress environments. We consider the regulatory network controlling expression of the multiple antibiotic resistance activator MarA, which regulates many downstream genes that confer tolerance to antibiotics and other inhibitors. Previous studies have shown that expression of MarA can be induced by compounds like salicylate or through mutations that eliminate transcriptional repression of the *marRAB* operon [19], [61]. However, the regulatory topology that controls expression of *marRAB* consists of a pair of interlinked positive and negative feedback loops, begging the question what role this additional regulatory structure provides, given that simple negative feedback would be sufficient to allow for inducible expression of MarA. Using a stochastic mathematical model, we studied the role of the feedback loops both separately and in combination. Our findings suggest that the negative feedback loop alone can produce inducible expression of MarA that exhibits low amplitude variability when both MarR_2_ molecules unbind from the promoter. Positive feedback serves to amplify this effect, creating stochastic pulses in MarA. Furthermore, we find that the nominal system parameters derived from the literature place the *marRAB* network in a regime with high variability and low cost. Thus, individual cells exhibit noisy MarA expression without an undue burden from expression of taxing resistance mechanisms.

Phenotypic heterogeneity in isogenic populations can provide a strategy for survival in uncertain environments. Our modeling results predict that MarA expression exhibits stochastic pulsing when uninduced. This variability, as measured using the coefficient of variation and noise strength, decreases as the system is induced. In the induced state there is little need for variability and it may be detrimental, causing some cells to move into a regime with low stress tolerance or unnecessary cost. Controlling for the mean levels of MarA expression and the timing of induction, we compared the fitness of two similar *marRAB* regulatory networks with differing levels of noise. We found that under constant conditions, it is disadvantageous to have variable MarA expression; in contrast, when stress profiles are dynamic, increased variability places a fraction of the population in a state that can tolerate the sudden appearance of a stressor such as an antibiotic.

There are several possible extensions to the findings presented here. For example, the cost of expressing MarA when the system is induced has an impact on the growth rate. Previous studies have shown that this affects processes such as protein dilution, transcription, and gene dosage [62], [63], all of which will have an impact on the system dynamics by introducing an additional indirect source of feedback. Other significant sources of feedback may also arise from changes in the nutrient environment or in expression of the proteolytic degradation machinery. Empirical growth laws, such as those presented in [62], [63], could be used to extend the model to account for these growth rate effects. In addition, it would be interesting to include the contributions of MarA homologs SoxS and Rob in our model to examine how crosstalk between the regulators affects the dynamics of MarA [14], [64]. Future studies to test our modeling predictions *in vivo*, are also of immediate interest. For example, a reporter for MarA could be used to measure the dynamics of expression at the single-cell level. These results could be compared to a synthetic gene network that exhibits external equivalence to the wildtype system, such as a network with only negative feedback that has the same dynamic range and induced MarA levels.

Populations of isogenic cells can exhibit phenotypic heterogeneity through a variety of dynamic processes. In our model of the *marRAB* network we observe stochastic pulsing without induction, but decreased variability after expression of MarA is induced. Allowing for tunable stochasticity can provide a flexible approach to stress tolerance. This strategy of integrating dynamic behaviors may prove to be a general mechanism for hedging against environmental uncertainty while allowing for well-defined sensory mechanisms that behave in a deterministic fashion.

## Methods

### Mathematical model

An exact, stochastic model was implemented using the Gillespie algorithm [46] and custom analysis code. Models are based on the processes described below, where the reaction rates and parameters are detailed and referenced in Text S1 and Table S1. The model treats cell growth and division implicitly unless otherwise noted, however results are similar when cell growth and division are explicitly modeled (Text S1, Fig. S7).

#### Promoter dynamics

Binding and unbinding of MarA and MarR_2_ to the *marRAB* promoter are modeled using:
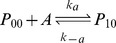


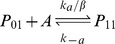


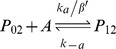


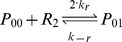


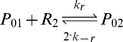


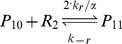


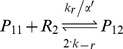
where P_ij_ represents the state of the promoter region, with i ∈ {0, 1} with A (MarA) molecules bound and j ∈ {0, 1, 2} with R_2_ (MarR_2_) molecules bound.

The association and dissociation rates take into consideration the number of binding sites and the competition in the binding between MarA and MarR_2_ (α, α′, β, and β′). Further details are provided in Text S1.

#### Transcription, translation, protein folding and MarR_2_ dimerization



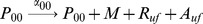


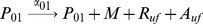


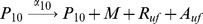


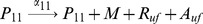


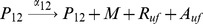


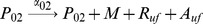


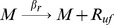


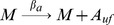


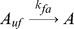


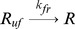


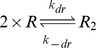
where M is mRNA, the subscript *uf* stands for the unfolded form of the protein, and R and R_2_ are the monomeric and dimeric forms of MarR.

Translation in bacteria occurs concurrently with transcription [65]. The model includes chemical reactions that couple transcription and translation to account for this. MarA promoter binding produces a fixed increase in the transcription rate, while MarR_2_ binding is assumed to dramatically decrease the transcription rate. MarR_2_ has very low translation efficiency relative to MarA [19]. MarA and MarR_2_ folding rates, k_fa_ and k_fr_, are assumed to be fast as a result of their small molecular weights (129 and 144 aa) [66] and the coupling of this process with translation *in vivo* [65]. MarR dimerization is also modeled. Further details are provided in Text S1.

#### Degradation



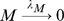


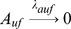


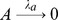


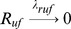


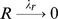


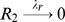



The mRNA transcript degradation rate, λ_M_, is constant, giving rise to an exponential decay. Similarly, protein degradation rates, λ_a_ and λ_r_, are fixed; the unfolded molecules are assumed to be degraded at the same rate as MarR and MarR_2_, however, since unfolded protein levels are low, changes to this parameter have little effect on the results. Because the dissociation rates for MarA and MarR_2_ are higher than the degradation rates (Table S1), we neglect protein degradation when the proteins are bound to the promoter.

#### Salicylate inhibition of MarR_2_

Since extracellular salicylate concentrations are 10^5^–10^6^ times higher than MarR_2_ concentrations, with typical values in our simulations varying from 1–25 nM for MarR_2_ (650–16,000 molecules/cell) and 0–10 mM for salicylate, the intracellular salicylate concentration is assumed to be several orders of magnitude greater than MarR_2_ and the reaction 
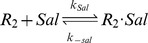
 is modeled as the pseudo-first-order chemical reaction:
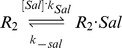



The two constants, k_sal_ and k_−sal_, were fit using experimental data from previous studies [5], [12] (Fig. S4). Given the low number of monomeric molecules, binding of salicylate with MarR was neglected.

### Feedback variants and feedback strength

Four alternative feedback combinations were created. To allow for a controlled comparison, we fixed the mean expression of MarA such that it was the same for all networks when the systems were uninduced (0 mM salicylate). This was achieved by modifying the transcription rate and MarA degradation rates, maintaining the parameters in realistic ranges. Specifically, the differences between the four alternatives are: (i) *Wildtype*: The operator region contains two identical binding sites for MarR_2_ and one for MarA. A total of six promoter states are modeled with distinct association and dissociation rates (see promoter dynamics above). (ii) *Only Positive*: Both binding sites for MarR_2_ are eliminated (k_r_ = 0). Thus, only the promoter states P_00_ and P_10_ are included in this model. (iii) *Only Negative*: The binding site for MarA is eliminated (k_a_ = 0). The promoter states P_00_, P_01_, and P_02_ remain. (iv) *No Feedback*: The system has basal, constitutive expression (k_r_ and k_a_ = 0). P_00_ is the only promoter state available. Details on parameters are given in Text S1.

### Heat maps of cost and noise strength

For all heat maps, the positive feedback loop strength and the negative feedback loop strength were systematically varied by modifying k_a_ and k_r_ in a range wide enough to include slow and fast transitions between promoter states. For each point, we calculated the noise strength and cost, using the average of six independent replicates.

The cost and noise strength were calculated based on MarA levels, using data generated after the initial system transients. The cost was calculated by using the function from [5] (given by *MCost* below), calculating equivalent salicylate to MarA levels using data from the Hill function in Fig. S4.

### Number of MarA pulses

To calculate the number of pulses in MarA, we used the following heuristic: a pulse was defined as a period when MarA levels exceeded 2/3 of the 90^th^ percentile of the number of molecules for at least 20 minutes. Pulses separated by less than 15 minutes were combined into a single pulse.

### Reduced noise variant

We created a variant with the same MarA expression for all salicylate concentration, but which had reduced noise compared to the *Wildtype* network. To achieve this, the inhibition constants, c_Inh1_ and c_Inh2_, were decreased, causing higher minimum MarR_2_ levels, increasing the probability of binding to the promoter and stopping the pulse at an earlier stage. In contrast to the *Wildtype* model, independent binding by MarA and MarR_2_ at the promoter is modeled, allowing MarR_2_ to bind easily when MarA is bound to the promoter. The parameters modified for this variant are detailed in Table S3.

### Stochastic competitive growth assay

To compare the two variants (*Wildtype* and *Reduced Noise*) in a head-to-head fashion, we simulated a competitive growth environment in the presence and absence of antibiotics. The following procedure was used to model competitive growth:

Step 1. Allow cells to grow without competition for an initialization period. Step 2: Simulate all individual cells for a fixed time, given an identical antibiotic time course. Step 3: Calculate the cost using the MarA levels for the cell. The cost of growing for the cell is the sum of the cost of expressing the resistance machinery, measured as a function of salicylate, and the cost of growing with the antibiotic, minus their product (Bliss independence is assumed [5]). The effective concentration of the antibiotic is inversely related to the concentration of salicylate [5]. For our experiments we used an compound that has the same cost for the cell as tetracycline and induces MarA with the same strength as salicylate, where both relations are defined in [5]. Cells with costs above a threshold are determined to be dead and eliminated from the competition. Competitive growth results are not sensitive to the exact value of this threshold. Step 4: Calculate, for each cell, the number of replications and replace underperforming cells with those that are growing well. Here, we take into account cell growth and division such that cells with lower costs are more prevalent than those with high costs. The number of daughter cells for each variant is obtained and the ratio between variants is calculated. This ratio is used to set the proportion between variants in the new population. In other words, dead and underperforming cells are replaced by healthy cells such that the new proportion between populations is the same as the proportion between the growth of the old populations. This allows us to maintain a constant number of cells, while at the same time representing the growth of the total population. This process is then repeated by returning to Step 2 until a variant overtakes the population or a predetermined maximum number of rounds is reached. Further details are provided in Text S1.

Three competitive growth simulations were performed: (1) Growth in a fluctuating environment: After the initialization period, an antibiotic profile is selected randomly. At each round, the antibiotic was either “on” or “off”, with the probability of antibiotic being present equal to 0, 0.25, 0.5, 0.75, or 1 for different simulations. A round corresponds to 540 min in the absence of antibiotic and 75 minutes in its presence. If the antibiotic was “on”, concentrations were selected randomly using an exponential distribution with a mean of 3 mM and maximum concentration of 4.5 mM. (2) Growth in a constant environment: The antibiotic concentration was kept fixed for both the initialization period and the competition simulations. (3) Fraction of surviving cells after a pulse of antibiotic: Only Steps 1, 2, and 3 of the algorithm described above are performed. After an initialization period in the absence of antibiotic, a pulse of antibiotic is introduced. The number of surviving cells, as measured by calculating those with cost of MarA and antibiotic to be below the predetermined threshold, are calculated for each simulation. Further details on the growth assay simulations are given in Text S1.

### Cost of growing with MarA

The cost of growing with salicylate is defined in [5] as:
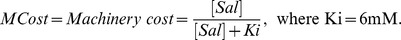
From the Hill function shown in Fig. S4 we obtain a relationship between MarA and salicylate concentration. Organizing the terms and assuming a maximum MarA concentration of 10,000 molecules/cell we find:
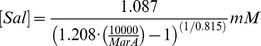
These two equations are combined to obtain the machinery cost.

### Cost of growing in the presence of antibiotics

Expression of the *mar* regulon genes provides antibiotic resistance, an effect that can be modeled as a reduction of the intracellular concentration of antibiotic:
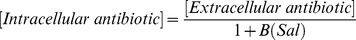
where B(Sal) is defined as in [5]:
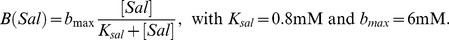



The cost of growing in the presence of antibiotic is modeled as in [5]:
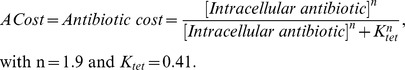



In our computations, a compound with the same cost function as tetracycline was used. This function is similar for chloramphenicol, with n = 1.97 and K_c_ = K_tet_ [5].

### Total cost

The total cost is assumed to be Bliss independent, as described in [5]:
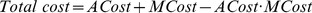



## Supporting Information

Figure S1 Stability analysis from the deterministic model. (A) Nullclines showing dMarA/dt = 0 and dMarR_2_/dt = 0 for 0, 1, 2.5, and 5 mM salicylate. (B) Stability of the equilibrium point at 0 mM salicylate and (C) 5 mM salicylate as a function of feedback loop strengths. Note that the equilibrium point is stable for all values of k_a_ and k_r_ shown.
(EPS)

Figure S2 Mean MarA levels as a function of salicylate concentration for the four feedback variants. The *Only Positive* and *No Feedback* systems are not responsive to salicylate as they lack negative feedback and their lines fall on top of each other. The *Wildtype* and *Only Negative* systems are responsive to salicylate and their lines coincide. Error bars show standard deviation over 100 simulations.
(EPS)

Figure S3 MarA histograms for the four feedback variants and the *Reduced Noise* network.
(EPS)

Figure S4MarA as a function of salicylate concentration. Experimental data from [5], [12] were used to tune the salicylate inhibition rate in the model and data were fit to a Hill function, as shown. The Hill function shown follows the equation: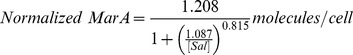.(EPS)

Figure S5 Parametric sensitivity analysis. For each parameter, simulations were run with that parameter at 1/2 the nominal value or 2 times the nominal value, with 0 and 5 mM salicylate. (A) Number of MarA pulses in 3000 minute simulations and the (B) noise strength of MarA. Sensitivity analysis for “k_r_ and k_-r_”and “k_a_ and k_-a_” vary both parameters together, such that their ratio remains constant. Error bars shown standard deviation over 5 simulations in (A) and 50 simulations in (B).
(EPS)

Figure S6 Stochastic simulations of the (A) uninduced *Wildtype* network and (B) uninduced *Reduced Noise* network. (C) MarA mean levels as a function of salicylate for the two networks. (D) Coefficient of variation and (E) noise strength of MarA for the two networks. Note the decrease in CV and noise strength for the *Reduced Noise* network relative to *Wildtype*. Error bars in (C)–(E) show standard deviation over 100 simulations.
(EPS)

Figure S7 Explicit cell growth and division model. (A) Number of proteins. The sharp drops correspond to cell division events. (B) Protein concentration given by number of proteins normalized by cell volume. Simulations show the *Wildtype* network with 0 mM salicylate.
(EPS)

Table S1 Model parameters.
(PDF)

Table S2 Modified parameters for the four feedback variants.
(PDF)

Table S3 Modified parameters for *Wildtype* and *Reduced Noise* networks.
(PDF)

Text S1 Supplementary information.
(PDF)
