# Supplementary material for: Tunable Stochastic Pulsing in the Escherichia coli Multiple Antibiotic Resistance Network from Interlinked Positive and Negative Feedback Loops
Source: PLoS Comput Biol. 2013 Sep 26;9(9):e1003229. doi: 10.1371/journal.pcbi.1003229 (PMC3784492; doi:10.1371/journal.pcbi.1003229)
Supplement: Table S1 — Model parameters. (PDF) [file pcbi.1003229.s008.pdf]

Table S1: Model parameters

		Value	Reaction	Propensity	References and Comments
k_{-a}	MarA-promoter dissociation rate	$1.8\ (\min)^{-1}$	$P_{10} \xrightarrow{k_{-a}} P_{00} + A$<br>$P_{11} \xrightarrow{k_{-a}} P_{01} + A$<br>$P_{12} \xrightarrow{k_{-a}} P_{02} + A$	$k_{-a} \cdot P_{10}$<br>$k_{-a} \cdot P_{11}$<br>$k_{-a} \cdot P_{12}$	Other feedback operons: 1.8 $\min^{-1}$ [1], 2.4 $\min^{-1}$ [2].<br>The stochastic pulsing is not disrupted for a wide range of k_{-a} and k_{-r} values.
k_a	MarA-promoter association rate	$\frac{k_{-a}}{1500}$ $(\text{molecules} \cdot \min)^{-1}$	$P_{00} + A \xrightarrow{k_a} P_{10}$<br>$P_{01} + A \xrightarrow{k_a/\beta} P_{11}$<br>$P_{02} + A \xrightarrow{k_a/\beta'} P_{12}$	$\frac{1}{V} \cdot k_a \cdot P_{00} \cdot A$<br>$\frac{1}{V} \cdot \frac{k_a}{\beta} \cdot P_{01} \cdot A$<br>$\frac{1}{V} \cdot \frac{k_a}{\beta'} \cdot P_{02} \cdot A$	Dissociation constant for MarA = 25nM = 1500 molecules/cell [3].<br>V is the ratio between the volume of the cell at a given time and the average volume of *E. coli* considered in the paper ($1\ \mu m^3$).
k_{-r}	$MarR_2$-promoter dissociation rate	$1.8\ (\min)^{-1}$	$P_{01} \xrightarrow{k_{-r}} P_{00} + R_2$<br>$P_{02} \xrightarrow{2 \cdot k_{-r}} P_{01} + R_2$<br>$P_{11} \xrightarrow{k_{-r}} P_{10} + R_2$<br>$P_{12} \xrightarrow{2 \cdot k_{-r}} P_{11} + R_2$	$k_{-r} \cdot P_{01}$<br>$2 \cdot k_{-r} \cdot P_{02}$<br>$k_{-r} \cdot P_{11}$<br>$2 \cdot k_{-r} \cdot P_{12}$	Other feedback operons: 1.8 $\min^{-1}$ [1], 2.4 $\min^{-1}$ [2].<br>The stochastic pulsing is not disrupted for a wide range of k_{-a} and k_{-r} values.
k_r	$MarR_2$-promoter association rate	$\frac{k_{-r}}{150}$ $(\text{molecules} \cdot \min)^{-1}$	$P_{00} + R_2 \xrightarrow{2 \cdot k_r} P_{01}$<br>$P_{01} + R_2 \xrightarrow{k_r} P_{02}$	$\frac{1}{V} \cdot 2 \cdot k_r \cdot P_{00} \cdot R_2$<br>$\frac{1}{V} \cdot k_r \cdot P_{01} \cdot R_2$	Dissociation constant for $MarR_2$ = 2.5nM = 150 molecules/cell [4-6].

			$P_{10} + R_2 \xrightarrow{2 \cdot k_r/\alpha} P_{11}$ $P_{11} + R_2 \xrightarrow{k_r/\alpha'} P_{12}$	$\frac{1}{V} \cdot \frac{2 \cdot k_r}{\alpha} \cdot P_{10} \cdot R_2$ $\frac{1}{V} \cdot \frac{k_r}{\alpha'} \cdot P_{11} \cdot R_2$	
α_{00}	Transcription rate with no $MarR_2$ molecules and no MarA molecules bound to the promoter	$0.40\,(\min)^{-1}$	$P_{00} \xrightarrow{\alpha_{00}} P_{00} + M + R_{uf} + A_{uf}$	$\alpha_{00} \cdot P_{00}$	From other systems: 0.36 min^{-1} [1], 0.1 min^{-1} [2]. <br>α_{00}, $\beta_{(a/r)}$ and c_{Act} are chosen to match the experimental data (10,000 molecules if $MarR_2$ binding sites are eliminated, 500 molecules in the basal level – See Supplementary Methods).
α_{01}	Transcription rate with one $MarR_2$ molecule and no MarA molecules bound to the promoter	$\alpha_{00}/\ c_{Inh1}\,(\min)^{-1}$	$P_{01} \xrightarrow{\alpha_{01}} P_{01} + M + R_{uf} + A_{uf}$	$\alpha_{01} \cdot P_{01}$	One molecule of $MarR_2$ is bound; the transcription rate is modified by c_{Inh1}.
α_{10}	Transcription rate with no $MarR_2$ molecules and one MarA molecule bound to the promoter	$\alpha_{00} \times c_{Act}\,(\min)^{-1}$	$P_{10} \xrightarrow{\alpha_{10}} P_{10} + M + R_{uf} + A_{uf}$	$\alpha_{10} \cdot P_{10}$	One molecule of MarA is bound; the transcription rate is modified by c_{Act}.
α_{11}	Transcription rate with one $MarR_2$ molecule and one MarA molecule bound to the promoter	$\alpha_{00} \times c_{Act} / c_{Inh1}\,(\min)^{-1}$	$P_{11} \xrightarrow{\alpha_{11}} P_{11} + M + R_{uf} + A_{uf}$	$\alpha_{11} \cdot P_{11}$	One molecule of MarA and one molecule of $MarR_2$ are bound; the transcription rate is modified by c_{Act} / c_{Inh1} .
α_{12}	Transcription rate with two $MarR_2$ molecules and one MarA molecule bound to the promoter	$\alpha_{00} \times c_{Act} /(c_{Inh1} \times c_{Inh2})$ $(\min)^{-1}$	$P_{12} \xrightarrow{\alpha_{12}} P_{12} + M + R_{uf} + A_{uf}$	$\alpha_{12} \cdot P_{12}$	One molecule of MarA and two molecules of $MarR_2$ are bound; the transcription rate is modified by c_{Act} / ($c_{Inh1} \times c_{Inh2}$).

α_{02}	Transcription rate with two $MarR_2$ molecules and no MarA molecules bound to the promoter	$\alpha_{00}/(c_{Inh1} \times c_{Inh2})\ (\min)^{-1}$	$P_{02} \xrightarrow{\alpha_{02}} P_{02} + M + R_{uf} + A_{uf}$	$\alpha_{02} \cdot P_{02}$	Two molecules of $MarR_2$ are bound; the transcription rate is modified by 1 / ($c_{Inh1} \times c_{Inh2}$).
c_{Act}	Activation factor	80			From another system: 20 [1].<br><br>*marRAB* is expressed strongly after induction [7]. α_{00}, $\beta_{(a/r)}$ and c*Act* are chosen to match experimental data from this study (10,000 molecules if $MarR_2$ binding sites are eliminated, 500 molecules in the basal level – See Supplementary Methods).
c_{Inh1}	Repression factor for the first $MarR_2$ binding	800			$MarR_2$ binding impairs RNA polymerase binding and progression [5,8]. The system is robust to changes in this parameter.
c_{Inh2}	Repression factor for the second $MarR_2$ binding	10			$MarR_2$ binding impairs RNA polymerase binding and progression [5,8]. The system is robust to changes in this parameter.
λ_M	mRNA degradation rate	$\ln(2)/24\ (\min)^{-1}$	$M \xrightarrow{\lambda_M} 0$	$\lambda_M \cdot M$	[9],Stochastic pulsing behavior is robust to changes in this parameter.
β_a	*marA* translation rate	$0.34 \times 20\ (\min)^{-1}$	$M \xrightarrow{\beta_a} M + A_{uf}$	$\beta_a \cdot M$	Translation rate = 34% of the *lacZ* translation rate [7].<br><br>*lacZ* translation rate: 18.8 min^{-1} [10], *lacZ* initialization every 3.2 sec [11].
β_r	*marR* translation rate	$0.044 \times 20\ (\min)^{-1}$	$M \xrightarrow{\beta_r} M + R_{uf}$	$\beta_r \cdot M$	Translation rate = 4.4% of the *lacZ* translation rate. [7].
k_{fa}	MarA folding rate	$5\ (\min)^{-1}$	$A_{uf} \xrightarrow{k_{fa}} A$	$k_{fa} \cdot A_{uf}$	Fast, due to the small size of the protein and the coupling of this process with translation *in vivo* [12]. The system is robust to changes in this value.

					Other examples: 60 min^{-1} for Cytochrome C [13], 0.9 min^{-1} for the synthetic oscillator [1].
k_{fr}	MarR folding rate	$5\,(\min)^{-1}$	$R_{uf} \xrightarrow{k_{fr}} R$	$k_{fr} \cdot R_{uf}$	Fast, due to the small size of the protein and the coupling of this process with translation *in vivo* [12]. The system is robust to variations in this value.<br><br>Other examples: 60 min^{-1} for Cytochrome C [13], 0.9 min^{-1} for the synthetic oscillator [1].
k_{dr}	MarR dimerization rate	$0.01\,(\text{molecules} \cdot \min)^{-1}$	$2 \times R \xrightarrow{k_{dr}} R_2$	$\frac{1}{V} \cdot k_{dr} \cdot \frac{R \cdot (R-1)}{2}$	Assumed to be consistent with the cI protein in *E. coli*, 0.01 mol^{-1} min^{-1} [14]. The system is robust to variations in this value.<br><br>Another example: 0.18 min^{-1} for the synthetic oscillator [1].
k_{-dr}	$MarR_2$ dimer disruption rate	$k_{dr}/50\,(\min)^{-1}$	$R_2 \xrightarrow{k_{-dr}} 2 \times R$	$k_{-dr} \cdot R_2$	Assumed to be consistent with the cI protein in *E. coli*, k_{dr} /50 min^{-1}. The system is robust to variations in this value.<br><br>Other example: k_{dr} /100 min^{-1} for the synthetic oscillator [1].
λ_{auf}	Unfolded MarA degradation rate	$\lambda_r\,(\min)^{-1}$	$A_{uf} \xrightarrow{\lambda_{auf}} 0$	$\lambda_{auf} \cdot A_{uf}$	We assumed only folded MarA to be actively degraded by Lon protease [15], setting the degradation rate for unfolded MarA at a reduced level, comparable to what we used for degradation of the folded $MarR_2$ protein. There is only a small amount of unfolded protein; as a result, the system is robust to variations in this parameter.
λ_a	MarA degradation	$\ln(2)\ (\min)^{-1}$	$A \xrightarrow{\lambda_a} 0$	$\lambda_a \cdot A$	[15]
λ_{ruf}	Unfolded MarR degradation rate	$\lambda_r\,(\min)^{-1}$	$R_{uf} \xrightarrow{\lambda_{ruf}} 0$	$\lambda_{ruf} \cdot R_{uf}$	We assumed the same degradation rate as the folded $MarR_2$ protein. There is only a small amount of unfolded protein; as a result, the system is robust to variations in this parameter.

λ_r	MarR and $MarR_2$ degradation	$\ln(2)/24\ (\text{min})^{-1}$	$R \xrightarrow{\lambda_r} 0$ $R_2 \xrightarrow{\lambda_r} 0$	$\lambda_r \cdot R$ $\lambda_r \cdot R_2$	We assumed the protein to be stable, using a degradation time that matches the dilution rate due to cell division in rich medium [16]. Changes in the parameter do not disrupt stochastic pulsing.
k_{sal}	$MarR_2$ allosteric inhibition rate	20 $(\text{molecules} \cdot \text{min})^{-1}$	$Sal + MarR_2 \xrightarrow{k_{Sal}} MarR_2 - Sal$	$\frac{1}{V} \cdot k_{Sal} \cdot MarR_2 \cdot Sal$	Selected to fit experimental data from [17] and [18]. The system is robust to changes in this parameter.
k_{-sal}	$MarR_2$-Salicylate complex disruption rate	0.5 $(\text{min})^{-1}$	$MarR_2 - Sal \xrightarrow{k_{-Sal}} Sal + MarR_2$	$k_{-Sal} \cdot MarR_2 - Sal$	Selected to fit experimental data from [17] and [18]. The system is robust to changes in this parameter.
		As detailed in Supplementary Methods, the parameters given below are used to fit the model to the following experimental data: 3.3-fold repression with only one $MarR_2$ site active and a 20-fold repression with both sites active [7,19]; competition in the binding between MarA and $MarR_2$ [5], either by sliding block [20] or alignment of the marbox with the -35 box [8]; approximately 9,000 molecules with 5mM of salicylate induction [4] and maximum expression with salicylate of 10,000 molecules ([17] with the data from [4]); basal expression of 500 molecules (Maximum level / Repression fold with both sites active).			
α	Inhibition in the binding of the first molecule of $MarR_2$ when MarA and no $MarR_2$ are bound	1000	$P_{10} + R_2 \xrightarrow{2 \cdot k_r/\alpha} P_{11}$		
α'	Inhibition in the binding of the second molecule of $MarR_2$ when MarA and $MarR_2$ are bound	1.5	$P_{11} + R_2 \xrightarrow{k_r/\alpha'} P_{12}$		
β	Inhibition in the binding of MarA when one $MarR_2$ molecule is bound	1.5	$P_{01} + A \xrightarrow{k_a/\beta} P_{11}$		

β'	Inhibition in the binding of MarA when two $MarR_2$ molecules are bound	1.5	$P_{02} + A \xrightarrow{k_a/\beta'} P_{12}$		

References

1. Stricker J, Cookson S, Bennett MR, Mather WH, Tsimring LS, et al. (2008) A fast, robust and tunable synthetic gene oscillator. Nature 456: 516-519.
2. Stamatakis M, Zygourakis K (2011) Deterministic and stochastic population-level simulations of an artificial lac operon genetic network. BMC bioinformatics 12: 301.
3. Martin RG, Rosner JL (2011) Promoter discrimination at class I MarA regulon promoters mediated by glutamic acid 89 of the MarA transcriptional activator of Escherichia coli. Journal of bacteriology 193: 506-515.
4. Martin RG, Bartlett ES, Rosner JL, Wall ME (2008) Activation of the Escherichia coli marA/soxS/rob regulon in response to transcriptional activator concentration. Journal of molecular biology 380: 278-284.
5. Martin RG, Jair K-W, Wolf RE, Rosner JL (1996) Autoactivation of the marRAB Multiple Antibiotic Resistance Operon by the MarA transcriptional activator in Escherichia coli. Journal of bacteriology 178: 2216-2223.
6. Alekshun MN, Levy SB (1999) The mar regulon: multiple resistance to antibiotics and other toxic chemicals. Trends in microbiology 7: 410-413.
7. Martin RG, Rosner JL (2004) Transcriptional and translational regulation of the marRAB multiple antibiotic resistance operon in Escherichia coli. Molecular microbiology 53: 183-191.
8. Martin RG, Rosner JL (1997) Fis, an accessorial factor for transcriptional activation of the mar (multiple antibiotic resistance) promoter of Escherichia coli in the presence of the activator MarA, SoxS, or Rob. Journal of bacteriology 179: 7410-7419.
9. Selinger DW, Saxena RM, Cheung KJ, Church GM, Rosenow C (2003) Global RNA Half-Life Analysis in Escherichia coli Reveals Positional Patterns of Transcript Degradation. Genome research: 216-223.
10. Santillán M, Mackey MC, Zeron ES (2007) Origin of bistability in the lac Operon. Biophysical journal 92: 3830-3842.
11. Kennell D, Riezman H (1977) Transcription and translation initiation frequencies of the Escherichia coli lac operon. Journal of molecular biology 114: 1-21.
12. Alberts B, Johnson A, Lewis J, Raff M, Roberts K, et al. (2002) How Cells Read the Genome: From DNA to Protein. Molecular Biology of the Cell. 4th ed. New York: Garland Science.
13. Krishna MMG, Lin Y, Mayne L, Walter Englander S (2003) Intimate View of a Kinetic Protein Folding Intermediate: Residue-resololding and Unfolding Rates, Homogeneityved Structure, Interactions, Stability, F. Journal of Molecular Biology 334: 501-513.
14. Bundschuh R, Hayot F, Jayaprakash C (2003) The role of dimerization in noise reduction of simple genetic networks. Journal of theoretical biology 220: 261-269.
15. Griffith KL, Shah IM, E. Wolf R (2004) Proteolytic degradation of Escherichia coli transcription activators SoxS and MarA as the mechanism for reversing the induction of the superoxide (SoxRS) and multiple antibiotic resistance (Mar) regulons. Molecular Microbiology 51: 1801-1816.
16. Sezonov G, Joseleau-Petit D, D'Ari R (2007) Escherichia coli physiology in Luria-Bertani broth. Journal of bacteriology 189: 8746-8749.
17. Wood KB, Cluzel P (2012) Trade-offs between drug toxicity and benefit in the multi-antibiotic resistance system underlie optimal growth of E. coli. BMC systems biology 6: 48.
18. Cohen SP, Levy SB, Foulds J, Rosner JL (1993) Salicylate induction of antibiotic resistance in Escherichia coli: activation of the mar operon and a mar-independent pathway. Journal of bacteriology 175: 7856-7862.
19. Seoane AS, Levy SB (1995) Characterization of MarR, the repressor of the multiple antibiotic resistance (mar) operon in Escherichia coli. Journal of bacteriology 177: 3414-3419.
20. Hammar P, Leroy P, Mahmutovic A, Marklund EG, Berg OG, et al. (2012) The lac Repressor Displays Facilitated. Science 1595: 1595-1598.
