# Supplementary material for: Tunable Stochastic Pulsing in the Escherichia coli Multiple Antibiotic Resistance Network from Interlinked Positive and Negative Feedback Loops
Source: PLoS Comput Biol. 2013 Sep 26;9(9):e1003229. doi: 10.1371/journal.pcbi.1003229 (PMC3784492; doi:10.1371/journal.pcbi.1003229)
Supplement: Table S2 — Modified parameters for the four feedback variants. (PDF) [file pcbi.1003229.s009.pdf]

Table S2: Modified parameters for the four feedback variants

Parameter	*Wildtype*	*Only Positive*	*Only Negative*	*No Feedback*
k_a	wt	wt	0	0
k_r	wt	0	wt	0
$\alpha_{00}, \alpha_{01}, \alpha_{10}, \alpha_{11}, \alpha_{02}, \alpha_{12}$	wt	wt * c_1	wt * c_{2a}	wt * c_3
λ_a	wt	wt	wt * c_{2b}	wt
k_{sal}	wt	wt	$20 \cdot 8.5 \frac{[Salicylate]^{0.5}}{([Salicylate]^{0.5} + 3^{0.5})}$	wt
