# Supplementary material for: Tunable Stochastic Pulsing in the Escherichia coli Multiple Antibiotic Resistance Network from Interlinked Positive and Negative Feedback Loops
Source: PLoS Comput Biol. 2013 Sep 26;9(9):e1003229. doi: 10.1371/journal.pcbi.1003229 (PMC3784492; doi:10.1371/journal.pcbi.1003229)
Supplement: Table S3 — Modified parameters for Wildtype and Reduced Noise networks. (PDF) [file pcbi.1003229.s010.pdf]

Table S3: Modified parameters for *Wildtype* and *Reduced Noise* networks

Parameter	*Wildtype*	*Reduced Noise*
α	1000	1
α'	1.5	1
β	1.5	1
β'	1.5	1
c_{Inh1}	800	3.32
c_{Inh2}	10	3
k_{sal}	20	$20 \cdot 5.5 \frac{[Salicylate]^{0.75}}{([Salicylate]^{0.75} + 0.6^{0.75})}$
