# Supplementary material for: Tunable Stochastic Pulsing in the Escherichia coli Multiple Antibiotic Resistance Network from Interlinked Positive and Negative Feedback Loops
Source: PLoS Comput Biol. 2013 Sep 26;9(9):e1003229. doi: 10.1371/journal.pcbi.1003229 (PMC3784492; doi:10.1371/journal.pcbi.1003229)
Supplement: Text S1 — Supplementary information. (PDF) [file pcbi.1003229.s011.pdf]

SUPPLEMENTARY INFORMATION

Stability analysis using deterministic model

To study the dynamics of the system as a function of salicylate levels, we developed a deterministic model using ordinary differential equations derived from the chemical reactions in Table S1 (Supplementary Methods). We present the stability analysis here to support the stochastic simulation results and provide insight into the underlying dynamics of the system.

SUPPLEMENTARY METHODS

Stochastic model

The cell volume was assumed to be constant and equal to $1\ \mu m^3$, unless otherwise noted.

Competitive MarA and $MarR_2$ binding at *marRAB* promoter

Previous experimental results indicate that competition occurs between the binding of MarA and $MarR_2$ at the promoter [1,2]. MarA produces an alignment between the MarA binding site and the -35 RNA polymerase binding site [2], enhancing the polymerase association rate, which produces a steric hindrance with $MarR_2$, with binding sites between the -35 and -10 sequences and overlapping the SD sequence [2]. Thus, in the model we assume a high inhibition of $MarR_2$ binding when MarA is bound (α and α'). A previous study on the Lac repressor has shown that when there is an obstacle within 40 nucleotides of the LacI binding site the association rate of this repressor with the promoter is decreased [3]. In our system, one of the $MarR_2$ binding sites is 28 nucleotides away from the MarA site, therefore we included inhibition of MarA binding when $MarR_2$ is bound (β and β'). The specific values of α, α', β and β' are set to agree with the experimental data from [4] when one $MarR_2$ binding site is eliminated, with the basal levels calculated to be 20 times lower than the maximum number of molecules when the system is induced [4,5]. The maximum number of MarA proteins (10,000 molecules) was extrapolated from previous studies [6], given 9,000 molecules per cell present when the system is induced with 5mM salicylate [7].

Transcription, translation, protein folding and $MarR_2$ dimerization

MarA induction, c_{Act}, increases the transcription rate by a constant factor set to match experimental data for the induced system with 5mM salicylate, as described above. $MarR_2$ binding is assumed to dramatically decrease the transcription rate (c_{Inh1} and c_{Inh2}); in the repressed state, with $MarR_2$ bound, the RNA polymerase is highly impaired in its binding and progression [4]. Translational activities for MarA and $MarR_2$ are 34 and 4.4% of the translation activity for *lacZ*. The *lacZ* translation rate is assumed 20 min^{-1} (Table S1).

The system is robust to changes in transcription and translation rates, as changes in one rate can be compensated with changes in the opposite direction in the other rate to keep the maximum number of molecules fixed; consequently, the system dynamics are conserved for a wide range of balanced transcription and translation rates.

Dimerization and dimer disruption rates, k_{dr} and k_{-dr}, were assumed to be consistent with the cI dimerization rates in *E. coli* [8], nevertheless, the system is robust to variation in folding and dimerization parameters; 100-fold changes in the values do not have a high impact in noise or protein levels.

Feedback variants and feedback strength

To obtain the variants lacking one or both feedback loops, the parameters were modified as detailed in Table S2. For the *Only Negative* network, the transcription and MarA degradation rates were changed to allow for a controlled comparison, where the same steady-state is achieved for all salicylate concentration (external equivalence, Fig. S2) and all but two parameters remained unaltered (internal equivalence, Table S2) [9].

In the Table S2, wt is the nominal value of the parameters given in Table S1. Table entries describe how this parameter was modified in each of the four feedback variants. For example, for the rate k_a the *Wildtype* values are used for those variants that keep the positive feedback (i.e. *Wildtype* and *Only Positive*), and the parameter is set to zero for the other two variants. In the third row, all the transcription rates are multiplied by the constant specified in the table. The following parameters were set to obtain the same steady state MarA levels for the four variants when uninduced: $c_1 = 1/5.416$, $c_{2a} = 44.282$, $c_{2b} = 1/1.520$, $c_3 = 3.705$. All other model parameters remained the same.

Stochastic competitive growth assay

Algorithm

Step 1. Initialize cells by simulating 6-12 hours of growth, with or without antibiotics depending on the specific computational experiment (described below). The final state of the system and the levels of each reactant in the cell are stored to initialize the next simulation round.

Step 2: Simulate all individual cells for a fixed time (5, 75, or 540 minutes depending on the experiment, as described below), given an identical antibiotic time course, using as a start point the final state for that cell in the previous simulation round.

Step 3: Calculate the cost using the MarA levels for the cell.
The cost of expressing genes in the *mar* regulon (e.g. efflux pumps, membrane proteins, stress response genes) is calculated as described in the Methods, using MarA mean levels from every 10th data point to calculate the weighted average of the cost over the entire period.

Cells with total costs above the arbitrary threshold of 0.975 are determined to be dead. However, the results presented here are not sensitive to the exact value of the threshold. Changing the threshold moves the maximum concentration of antibiotic that cells are able to tolerate, but does not affect the overall trends observed.

Step 4: Using the total cost, the number of daughter cells derived from each individual cell is calculated by:

$$Final\ number\ of\ cells = 1 \cdot e^{\left(\frac{(1-cost)\cdot Time}{Duplication\ time}\right)}$$

A cell division time of 45 minutes is assumed, variations in this constant only change the time scale of the algorithm and have no impact on the results.

The cost calculated from a short simulation (75 minutes if antibiotic is present, or 540 minutes if it is not) is used to extrapolate the final number of cells after 12 hours if antibiotic is present, or 72 hours if it is not. These durations take into consideration the fact that a cell is more likely to grow in an antibiotic-free environment than when antibiotics are present. The extrapolation assumes that the distribution of costs for the population is constant over time.

The total number of daughter cells for each variant is obtained and the proportion of daughter cells for each

variant is used to set the levels in the new population. That is, the dead and underperforming cells are replaced by growing cells such that the new ratio between populations is the same as the final ratio in the previous population. This allows us to maintain a constant number of cells, while representing the growth of the total population.

Three classes of growth simulations were conducted. The details for each are described below.

Growth in a fluctuating environment: At each round, the antibiotic was either "on" or "off", with probability of being present equal to 0, 0.25, 0.50, 0.75 or 1 for different runs. If antibiotic was present, the concentration was drawn from an exponential distribution with mean of 3mM and maximum concentration of 4.5mM. Setting a maximum concentration avoids the occurrence of complete population extinction. 200 cells of each variant were simulated, starting with an initialization period of 12 hours of growth with no antibiotic. Next, we simulated 50 rounds of antibiotic stress. If one variant reached 100% of the population prior to 50 rounds, simulations were stopped since this variant would persist for the remaining rounds. For each round, every cell was simulated for 540 minutes in the absence of antibiotic or 75 minutes in the presence of antibiotic.

If antibiotic is present, an initial 5 minute simulation was run in order to determine which cells survived (those with costs below the 0.975 threshold). Here we assumed that if a cell is not able to accumulate enough MarA to grow with a cost below the threshold during the initial 5 minute period it will die before being able to respond to the environment change. Similar results are obtained if the threshold or the time span are varied, as long as the cells are not allowed to reach steady state. Following this 5 minute simulation, a 30 minute simulation was run to allow the surviving cells to reach steady state, then another 75 minute simulation was run at steady state to calculate the average cost for the cell. The average cost was used to calculate the number of replications in 12 hours (without antibiotic) or 72 hours (with antibiotic). Eight independent replicates for each probability of antibiotic addition were run.

Growth in a constant environment: The antibiotic concentration was kept fixed. 200 cells of each variant were simulated, with a 12 hour (if no antibiotic is present) or 6 hours (if antibiotic is present) initialization period. Over the course of 10 rounds every cell was simulated for 540 minutes (in the absence of antibiotic) or 75 minutes (in the presence) and average costs were calculated as indicated for the fluctuating environment. This average cost was used to calculate the number of replications in 12 hours (without antibiotic) or 72 hours (with antibiotic). Three independent replicates were run for each antibiotic level.

Fraction of surviving cells after a pulse of antibiotic: 10,000 cells of each variant were simulated. A pulse of antibiotic was introduced after a 12 hour initialization period with no antibiotic present. The surviving cells were those with cost below 0.975 after the 5 minute simulation. Five independent replicates were run.

Deterministic model

Applying the law of mass action, a description of the system based on the chemical reactions given in Table S1 was developed using ordinary differential equations:

$$\frac{dP_{00}}{dt} = P_{10} \cdot k_{-a} + P_{01} \cdot k_{-r} - P_{00} \cdot k_a \cdot A - 2 \cdot P_{00} \cdot k_r \cdot R_2$$

$$\frac{dP_{01}}{dt} = P_{00} \cdot 2 \cdot k_r \cdot R_2 + P_{11} \cdot k_{-a} + P_{02} \cdot 2 \cdot k_{-r} - P_{01} \cdot k_{-r} - P_{01} \cdot \frac{k_a}{\beta} \cdot A - P_{01} \cdot k_r \cdot R_2$$

$$\frac{dP_{10}}{dt} = P_{00} \cdot k_a \cdot A + P_{11} \cdot k_{-r} - P_{10} \cdot k_{-a} - P_{10} \cdot 2 \cdot {k_r}/{\alpha} \cdot R_2$$

$$\frac{dP_{11}}{dt} = P_{10} \cdot 2 \cdot {k_r}/{\alpha} \cdot R_2 + P_{01} \cdot {k_a}/{\beta'} \cdot A + P_{12} \cdot 2 \cdot k_{-r} - P_{11} \cdot k_{-r} - P_{11} \cdot k_{-a} - P_{11} \cdot {k_r}/{\alpha'} \cdot R_2$$

$$\frac{dP_{12}}{dt} = P_{11} \cdot {k_r}/{\alpha'} \cdot R_2 + P_{02} \cdot {k_a}/{\beta} \cdot A - P_{12} \cdot 2 \cdot k_{-r} - P_{12} \cdot k_{-a}$$

$$\frac{dP_{02}}{dt} = P_{01} \cdot k_r \cdot R_2 + P_{12} \cdot k_{-a} - P_{02} \cdot 2 \cdot k_{-r} - P_{02} \cdot {k_a}/{\beta} \cdot A$$

$$\frac{dM}{dt} = P_{00} \cdot \alpha_{00} + P_{01} \cdot \alpha_{01} + P_{10} \cdot \alpha_{10} + P_{11} \cdot \alpha_{11} + P_{12} \cdot \alpha_{12} + P_{02} \cdot \alpha_{02} - \mathrm{M} \cdot \lambda_M$$

$$\frac{dA_{uf}}{dt} = P_{00} \cdot \alpha_{00} + P_{01} \cdot \alpha_{01} + P_{10} \cdot \alpha_{10} + P_{11} \cdot \alpha_{11} + P_{12} \cdot \alpha_{12} + P_{02} \cdot \alpha_{02} + M \cdot \beta_a - A_{uf} \cdot \lambda_{auf} - A_{uf} \cdot k_{fa}$$

$$\frac{dR_{uf}}{dt} = P_{00} \cdot \alpha_{00} + P_{01} \cdot \alpha_{01} + P_{10} \cdot \alpha_{10} + P_{11} \cdot \alpha_{11} + P_{12} \cdot \alpha_{12} + P_{02} \cdot \alpha_{02} + M \cdot \beta_r - R_{uf} \cdot \lambda_{ruf} - R_{uf} \cdot k_{fr}$$

$$\frac{dA}{dt} = P_{10} \cdot k_{-a} + P_{11} \cdot k_{-a} + P_{12} \cdot k_{-a} + A_{uf} \cdot k_{fa} - P_{00} \cdot k_a \cdot A - P_{01} \cdot {k_a}/{\beta'} \cdot A - P_{02} \cdot {k_a}/{\beta} \cdot A - A \cdot \lambda_a$$

$$\frac{dR}{dt} = R_{uf} \cdot k_{fr} - R \cdot \lambda_r - R^2 \cdot k_{dr} + R_2 \cdot k_{-dr}$$

$$\frac{dR_2}{dt} = P_{01} \cdot k_{-r} + P_{11} \cdot k_{-r} + P_{02} \cdot 2 \cdot k_{-r} + P_{12} \cdot 2 \cdot k_{-r} + R^2 \cdot k_{dr}/2 - R_2 \cdot k_{-dr}/2 - P_{00} \cdot 2 \cdot k_r \cdot R_2$$
$$- P_{10} \cdot 2 \cdot {k_r}/{\alpha} \cdot R_2 - P_{01} \cdot k_r \cdot R_2 - P_{11} \cdot {k_r}/{\alpha'} \cdot R_2 - R_2 \cdot \lambda_{\mathrm{r}} - R_2 \cdot k_{sal} + R_2Sal \cdot k_{-sal}$$

$$\frac{dR_2Sal}{dt} = R_2 \cdot k_{sal} - R_2Sal \cdot k_{-sal} - R_2Sal \cdot \lambda_r$$

For nullcline calculations, the derivatives of all state variables were set to zero. Using Mathematica, we reduced the system into equations to obtain lines showing $\frac{dA}{dt} = 0$ and $\frac{dR_2}{dt} = 0$.

To evaluate stability of the equilibrium point we calculated the eigenvalues of the Jacobian for the full system of differential equations, evaluated at the equilibrium point.

Explicit Cell Growth Model

Unless otherwise noted, the models treat cell growth and division implicitly, including the dilution rate in the effective protein degradation rate and assuming that cells maintain a constant volume. To test whether this modeling assumption was valid, we conducted simulations that explicitly model cell growth for the *Wildtype* system with 0mM salicylate (Fig. S7). Model parameters and structure are the same as in the implicit growth model with the following changes: the protein dilution rate as set by cell division was subtracted from the

degradation rates given in Table S1 [10], cell volume was modeled as growing exponentially with time [11], and the propensity values were updated as a function of volume (Table S1). We assumed a cell division time of 24 minutes, based on the *E. coli* doubling time in log phase with rich medium [12]. At each cell division event, the cell volume was divided in two and the molecules were partitioned following a binomial distribution with probability 0.5 [13]. Explicit modeling of cell growth and division has little effect on the results. Importantly, pulses in the MarA are observed in models that treat cell growth and division both explicit and implicitly.

References

1. Martin RG, Jair K-W, Wolf RE, Rosner JL (1996) Autoactivation of the marRAB Multiple Antibiotic Resistance Operon by the MarA transcriptional activator in Escherichia coli. Journal of bacteriology 178: 2216-2223.
2. Martin RG, Rosner JL (1997) Fis, an accessorial factor for transcriptional activation of the mar (multiple antibiotic resistance) promoter of Escherichia coli in the presence of the activator MarA, SoxS, or Rob. Journal of bacteriology 179: 7410-7419.
3. Hammar P, Leroy P, Mahmutovic A, Marklund EG, Berg OG, et al. (2012) The lac Repressor Displays Facilitated. Science 1595: 1595-1598.
4. Martin RG, Rosner JL (2004) Transcriptional and translational regulation of the marRAB multiple antibiotic resistance operon in Escherichia coli. Molecular microbiology 53: 183-191.
5. Seoane AS, Levy SB (1995) Characterization of MarR, the repressor of the multiple antibiotic resistance (mar) operon in Escherichia coli. Journal of bacteriology 177: 3414-3419.
6. Wood KB, Cluzel P (2012) Trade-offs between drug toxicity and benefit in the multi-antibiotic resistance system underlie optimal growth of E. coli. BMC systems biology 6: 48.
7. Martin RG, Bartlett ES, Rosner JL, Wall ME (2008) Activation of the Escherichia coli marA/soxS/rob regulon in response to transcriptional activator concentration. Journal of molecular biology 380: 278-284.
8. Bundschuh R, Hayot F, Jayaprakash C (2003) The role of dimerization in noise reduction of simple genetic networks. Journal of theoretical biology 220: 261-269.
9. Savageau MA (2001) Design principles for elementary gene circuits: Elements, methods, and examples. Chaos 11: 142-159.
10. Alon U (July 7, 2006) An introduction to system biology: Design principles of biological circuits.: Chapman & Hall/CRC Mathematical & Computational Biology.
11. Cooper S (1988) Leucine uptake and protein synthesis are exponential during the division cycle of Escherichia coli B/r. Journal of bacteriology 170: 436-438.
12. Sezonov G, Joseleau-Petit D, D'Ari R (2007) Escherichia coli physiology in Luria-Bertani broth. Journal of bacteriology 189: 8746-8749.
13. Swain PS, Elowitz MB, Siggia ED (2002) Intrinsic and extrinsic contributions to stochasticity in gene expression. Proceedings of the National Academy of Sciences of the United States of America 99: 12795-12800.
